# Formation of Stoichiometric CsF*_n_* Compounds

**DOI:** 10.1038/srep07875

**Published:** 2015-01-22

**Authors:** Qiang Zhu, Artem R. Oganov, Qingfeng Zeng

**Affiliations:** 1 Department of Geosciences, Stony Brook University, Center for Materials by Design, Institute for Advanced Computational Science, Stony Brook University, NY 11794, USA; 2 Department of Problems of Physics and Energetics, Moscow Institute of Physics and Technology, 9 Institutskiy lane, Dolgoprudny city, Moscow Region, 141700, Russia; 3 School of Materials Science and Engineering, Northwestern Polytechnical University, Xi'an, 710072, China; 4 Science and Technology on Thermostructural Composite Materials Laboratory, Northwestern Polytechnical University, Xi'an, 710072, China

## Abstract

Alkali halides *MX*, have been viewed as typical ionic compounds, characterized by 1:1 ratio necessary for charge balance between M^+^ and X^−^. It was proposed that group I elements like Cs can be oxidized further under high pressure. Here we perform a comprehensive study for the CsF-F system at pressures up to 100 GPa, and find extremely versatile chemistry. A series of CsF*_n_* (*n* ≥ 1) compounds are predicted to be stable already at ambient pressure. Under pressure, 5*p* electrons of Cs atoms become active, with growing tendency to form Cs (III) and (V) valence states at fluorine-rich conditions. Although Cs (II) and (IV) are not energetically favoured, the interplay between two mechanisms (polyfluoride anions and polyvalent Cs cations) allows CsF_2_ and CsF_4_ compounds to be stable under pressure. The estimated defluorination temperatures of CsF*_n_* (*n* = 2,3,5) compounds at atmospheric pressure (218°C, 150°C, -15°C, respectively), are attractive for fluorine storage applications.

In general, for a given ionic compound A*_m_*B*_n_*, the stoichiometry reflects the ratio of valences (or the ratio of cationic and anionic formal charges). Yet, some ionic compounds do not strictly obey this rule. For instance, MgO_2_ can be prepared at very high oxygen fugacities, in which anions form the peroxide group [O_2_]^2−^ 1. The variation of stoichiometry comes from the formation of polyatomic anions (such as those in peroxides, superoxides^2^, polyiodides^3^, etc), without changing cation valences. It was found that peroxides (usually unstable or metastable) become thermodynamically stable under higher pressures^1^.

It appears that increasing pressure promotes the formation of increasing oxidation states. Our recent work has discovered that Xe will form stable oxides under high pressure, in which Xe exhibits the oxidation states of +2, +4 and +6^4^. In this case, the new stoichiometry is no longer from the anion-anion bonds (O-O), but from the higher valence of Xe. Elements around Xe in the Periodic Table are expected to undergo similar transitions. In particular, Cs, in the electronic configuration [Xe]6*s*^1^, is a natural choice to study this possibility. Indeed, Miao^5^ has recently reported that Cs, under pressure, can adopt oxidation states higher than +1 to form a series of stable CsF*_n_* compounds. According to Miao's calculation, CsF_2_ becomes stable at 5 GPa, CsF_3_ at 15 GPa, and CsF_5_ at 50 GPa. Considering [CsF_2_]^−^ and [CsF_5_] are isoelectronic to the well-known molecular XeF_2_ (Ref. 6) and [XeF_5_]^−^ (Ref. 7), this picture makes sense. However, Miao suggested that CsF_2_ and CsF_4_ adopt structures similar to those of XeF_2_ and XeF_4_, with the oxidation states of Cs being +2 and +4. In particular, an *I*4/*mmm* CsF_2_ was proposed to be thermodynamically stable at 10 GPa, which appears to break the isoelectronic analogy and involve Cs^2+^ ions isoelectronic to unknown and unstable Xe^+^ ion. To resolve this, we performed a comprehensive investigation of CsF*_n_* system under pressures up to 100 GPa. Our calculation uncovers quite a different scenario from Miao's report. We further explain that interplay between two mechanisms (polyfluoride anions and increase of Cs oxidation state) results in an unexpected variety of stable CsF*_n_* compounds under moderate pressure.

## Results and Discussion

**Figure 1:**
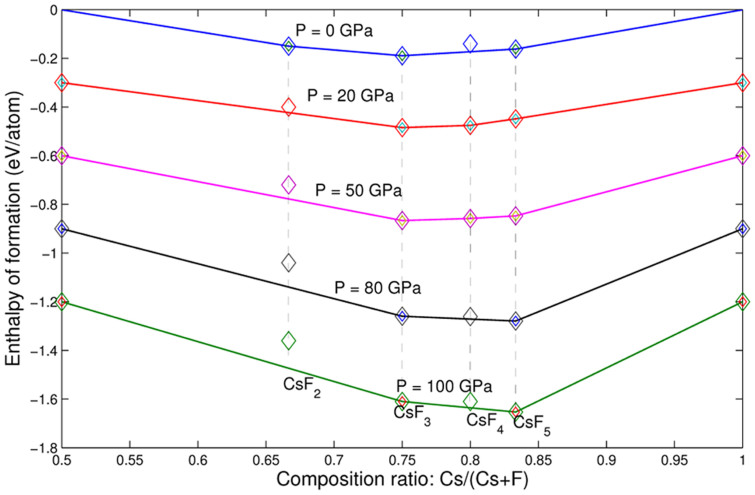
Convex hull diagrams of CsF*_n_* at different pressures.

We have performed variable-composition structure searches using the USPEX code^8^,^9^,^10^,^11^ with up to 24 atoms in the unit cell at pressures of 0 and 100 GPa for the Cs-F system, in which we found only CsF*_n_* (*n* ≥ 1) to be stable, with CsF being stable at all pressures. Thus we focused our search on the CsF-F system at pressures 0, 30, 50, 75, 100 GPa. As shown in Fig. 1, these searches yielded the correct crystal structures for CsF and F, and a series of polyfluoride compounds as stable states, including CsF_2_, CsF_3_, CsF_5_, which are thermodynamically stable already at ambient pressure. As pressure increases, CsF_2_ becomes unstable above 19 GPa, while CsF_4_ appears on the convex hull (i.e. is thermodynamically stable) at pressures in the range between 17–80 GPa. CsF_3_ and CsF_5_ are stable in the entire pressure range up to 100 GPa. However, all of the stable compounds undergo a series of phase transitions, with dramatic changes of the electronic structure.

**Figure 2:**
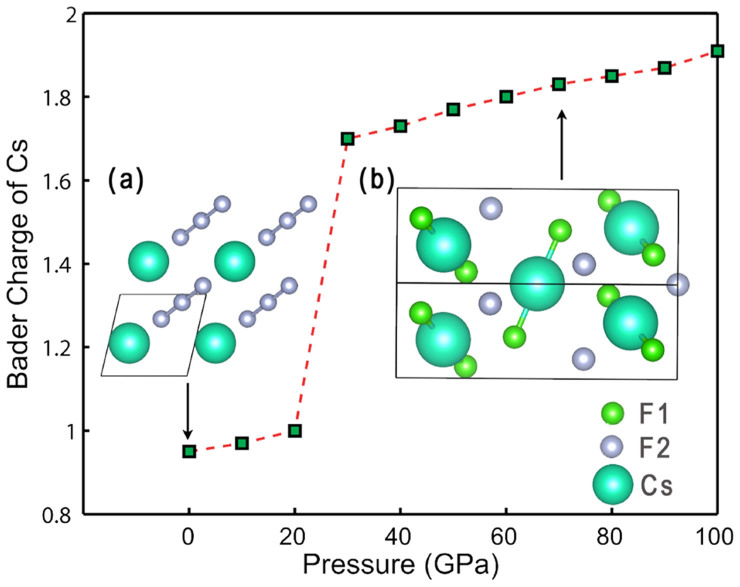
Bader charge of Cs in stable CsF_3_ compounds as a function of pressure. Insets (a), 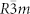 CsF_3_ structure at 0 GPa; (b) *C*2/*c* CsF_3_ structure at 70 GPa.

Let us first look at CsF_3_ phases. At ambient pressure, we find CsF_3_ adopts a rhombohedral structure (space group 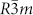), which is made of Cs^+^ and linear symmetric [F_3_]^−^ species. The F-F distance in the [F_3_] is 1.736 Å, indicating, as expected, weaker bonding than in the F_2_ molecule (F-F bond length 1.442 Å). Bader analysis also supports this conclusion: there is a charge transfer of 0.950 *e* from Cs to F_3_ species, very close to the value in CsF (0.928 *e*), but the charge distribution within F_3_ is not even: two end F atoms have the charge of -0.406 *e*, while the central atom only has -0.138 *e*. Trihalide anions [X_3_]^−^ (X = Br, I) are well known, but the [F_3_]^−^ species has only been experimentally found as CsF_3_ complexes in argon matrix^12^,^13^. Here, we for the first time report its existence in a thermodynamically stable crystalline phase. According to DFT calculation, 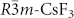 is stable against decomposition to CsF and F_2_ (the formation energy is about -0.189 eV/atom at T = 0 K, P = 1 atm). At 27 GPa, CsF_3_ undergoes a phase transition to a monoclinic phase *C*2/*c*. More interestingly, this structural transition coincides with a striking increase in Cs's Bader charge, as shown in Fig. 2. At 30 GPa, Cs has a charge of +1.7 *e*, far beyond the +1 formal charge of alkali elements under ambient conditions, suggesting Cs^+^ has been further oxidized. Our previous study has shown that Xe under high pressure can be oxidized to +2, +4 and +6 states. Cs^3+^ is isoelectronic to Xe^2+^. This phase transition can be interpreted as a transition from Cs^+^[F_3_]^−^ to [CsF_2_]^+^[F]^−^. This is also evidenced by the dramatic change of Cs-F distance. In the *C*2/*c* phase, there are two types of F atoms (F1 and F2), each Cs is surrounded by 2 F1 and 4 F2. At 50 GPa, the calculated Cs-F1 distance is 2.01 Å, while the Cs-F2 distance is 2.58 Å, appearing to be consistent with a Jahn-Teller distortion related to the open-shell Cs^3+^ configuration. However, compared with Cs-F bond length (2.69 Å) in ionic CsF, we conclude that Cs-F2 interaction is very close to a typical ionic Cs-F bond, while each individual Cs-F1 bond has much stronger interaction (more covalent bonding). Therefore, it can be viewed as [CsF_2_]^+^[F]^−^ complex. A similar discussion can be also found in Miao's work^5^. But his previously proposed *C*2/*m* structure is less stable than the *C*2/*c* structure found here.

**Figure 3:**
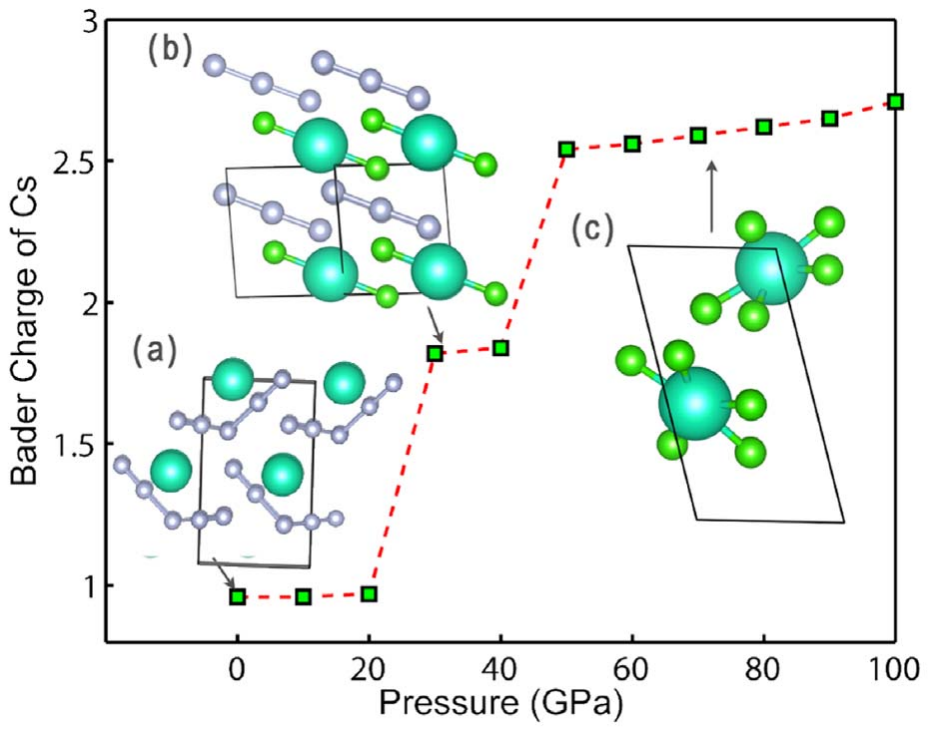
Bader charge of Cs in stable CsF_5_ compounds as a function of pressure. Insets (a), *P*2_1_-CsF_5_ structure at 0 GPa; (b) *C*2/*m*-CsF_5_ structure at 30 GPa; (c) *Fddd*-CsF_5_ structure at 70 GPa.

Similar to CsF_3_, CsF_5_ is also stable in the entire investigated pressure range between 0–100 GPa. At 0 GPa, we found a monoclinic *P*2_1_ phase is stable against decomposition to any other stable compositions (Cs, CsF, CsF_3_, F). *P*2_1_-CsF_5_ can be described as packing of Cs^+^ and [F_5_]^−^ species. [F_5_]^−^ ion has a V-shape and F-F bond lengths are 1.617, 1.953, 1.858, 1.617 Å, and F-F-F bond angle at central F atom is 98.592°. Bader analysis in Fig. 3 shows that the entire F_5_ group has charge -0.958 *e*. The hypothetical pentafluoride anion [F_5_]^−^ has also been proposed by Riedel^12^, and we confirm it can exist at ambient pressure in a stable compound. At 4 GPa, a new phase with [F_5_]^−^ groups and *C*2/*c* symmetry becomes stable. Around 21 GPa, *C*2/*c* phase transforms to another monoclinic *C*2/*m* phase, which can be represented as [CsF_2_] [F_3_]. The [CsF_2_] unit is very similar to the one in *C*2/*c*-CsF_3_ (Bader charge is 0.820*e*), indicating that Cs achieves the +3 oxidation state. At the same time, [F_3_] is a typical polyfluoride anion with Bader charge -0.820*e*; thus the whole structure can be viewed as [CsF_2_]^+^[F_3_]^−^. At 47 GPa, consistent with Miao's results^5^, we found a structure based on the packing of CsF_5_ molecules. We again plot the variation of Cs's Bader charge in stable CsF_5_ compounds with pressure. Indeed, analysis indicates a two-step oxidation of Cs +1 → +3 → +5, coinciding with the transition sequence (from *C*2/*c* to *C*2/*m* at 21 GPa, and from *C*2/*m* to *Fddd* at 47 GPa).

Our results show CsF_3_ and CsF_5_ are stable alongside the known compound CsF in the whole investigated pressure range (0–100 GPa). Unlike the recently discovered exotic sodium chlorides^14^, most of which are metallic, all of the predicted caesium fluorides are insulators. There are two factors determining the stoichiometry of these insulating compounds: (1) Cs's valence state transition (I → III → V); (2) formation of polyfluoride anions ([F_3_]^−^, [F_5_]^−^). Note that this is different from the previous study^5^, in which the latter factor was overlooked, along with a large number of stable phases. Due to these two competing mechanisms, one can expect other stoichiometries can be stabilized as well. Indeed, we found CsF_2_ and CsF_4_ can be stable at intermediate pressure ranges.

**Figure 4:**
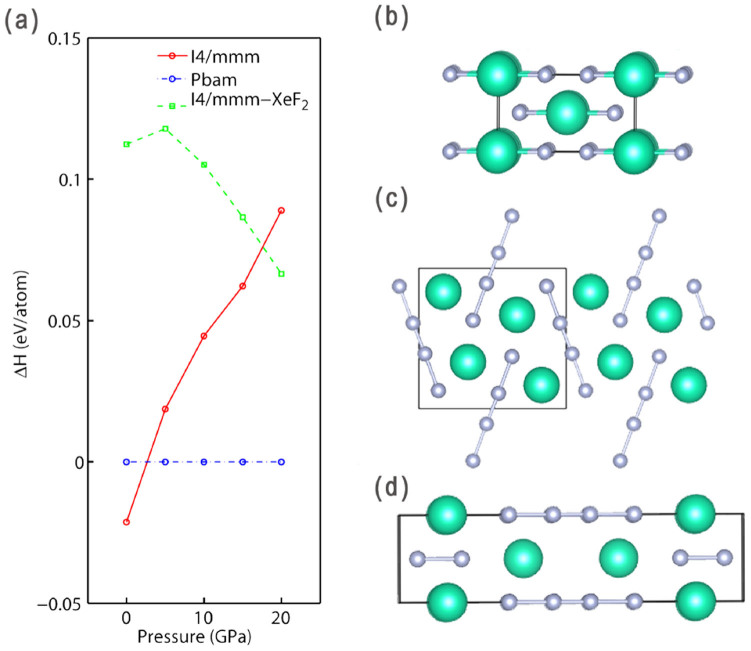
(a) Enthalpy of formation relative to *Pbam*-CsF_2_ as a function of pressure; (b) unstable molecular *I*4/*mmm*-CsF_2_ at 0 GPa; (c) stable *I*4/*mmm*-CsF_2_ structure (stable between 0-2.8 GPa); (d) *Pbam*-CsF_2_ structure (stable between 2.8-19 GPa).

**Figure 5:**
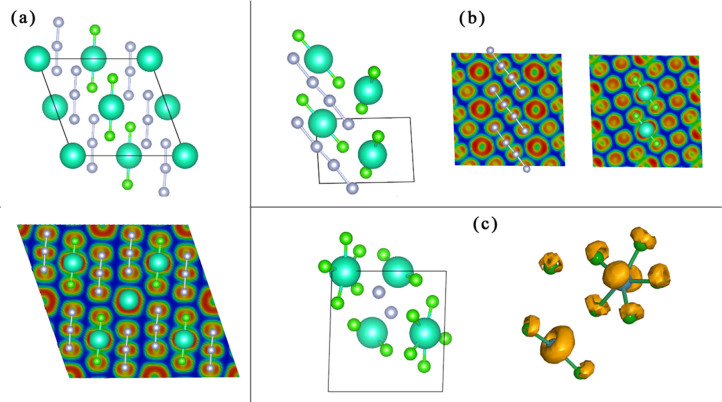
The stable crystal structures of CsF_4_ and their corresponding (sliced or isosurfaced) ELF pictures at pressures of (a) 20 GPa, (b) 50 GPa, (c) 80 GPa.

Previously, a tetragonal (*I*4/*mmm*) XeF_2_-like molecular structure was proposed to be stable at 5-20 GPa^5^. Our search found molecular CsF_2_ crystal to be unstable against decomposition to CsF_3_ and CsF at all pressures. A class of CsF_2_ compounds, however, has been found to be stable at low pressures in our prediction. At 0 GPa, another *I*4/*mmm* CsF_2_ phase is found to be stable. As shown in Fig. 4c, it consists of [Cs]^+^ and [F_4_]^2−^ ions. The calculated Bader charges are 0.924 *e* for [Cs], and -1.848 *e* for [F_4_], suggesting the formation of [Cs]^+^ and [F_4_]^2−^. Therefore, [Cs]^2+^ is not favoured by energy, but CsF_2_ can be stabilized due to the formation of the [F_4_]^2−^ anion. [F_4_]^2−^ has not been observed by chemists so far, except that Riedel et al^12^ theoretically investigated the possibility of [F_4_]^−^. Yet our comprehensive structural search suggests that [F_4_]^2−^ based CsF_2_ should be stable. Tetraiodide anion [I_4_]^2−^ is known^3^. Our results suggest that fluorine follows that same trend under high pressure. *I*4/*mmm*-CsF_2_ would undergo a phase transition to an orthorhombic phase (*Pbam*) at 2.8 GPa, which also contains Cs^+^ and [F_4_]^2−^ ions. Above 19 GPa, CsF_2_ is no longer stable as there is a dramatic change in the valence of Cs from (I) to (III). At around 20 GPa, CsF_4_ becomes stable in a monoclinic form (*C*2/*m*) (Fig. 5a). One can clearly see from the electron localization function (ELF) that half of Cs atoms have strong bonding with two neighbouring F atoms, and the other half of Cs atoms are simple Cs^+^ cations. The remaining F atoms form [F_3_]^−^ ions, as we already saw above in both CsF_3_ and CsF_5_. Thus, it can be viewed as [CsF_2_]^+^[Cs]^+^2[F_3_]^−^. Bader analysis also supports this interpretation. Half of Cs atoms have Bader charge of 1.049*e*, half of Cs have 1.773*e*. This suggests that Cs firstly achieves III valence state in CsF_4_. At 31 GPa, all Cs atoms are oxidized to +3 state (Bader charge 1.829*e*), and the structure has space group *P*-1. As shown in Fig. 5b, the whole structure can be represented as 2[CsF_2_]^+^[F_4_]^2−^ ([F_4_]^2−^ anions appear again!). Miao investigated the possibility of CsF_4_ molecule structurally similar to XeF_4_, which is contradictory to chemical intuition (CsF_4_ can be neither isostructural nor isoelectronic to XeF_4_). Indeed, our results suggest that Cs^4+^ based compound is energetically unfavored. But we found that the most stable CsF_4_ structure above 57 GPa shows the oxidation state higher than +3. As shown in Fig. 5c, it can be viewed as [CsF_2_]^+^[F]^−^ · [CsF_5_]^0^. Therefore, half of Cs atoms (in [CsF_2_]) have III valence, while the other half of Cs (in [CsF_5_]) have V valence state. The resulting structure, crystallizing in *P*-1 symmetry, is stable up to 79 GPa between *C*2/*c*-CsF_3_ and *Fddd*-CsF_5_. We note that there also exists a stable phase of XeF_3_ that can be represented as [XeF_2_]· [XeF_4_]^15^.

Light halogens, fluorine (F) and chlorine (Cl), at normal conditions are highly reactive and toxic gases. For chemical industry and laboratory use, this presents great inconvenience. Their storage in the gaseous form (even as liquefied gases) is very inefficient, and compressed gas tanks may explode, presenting great dangers. At normal conditions, the volume of 22.4 litres (L) of pure fluorine gas weighs just 36 grams (g), illustrating the dismal inefficiency of storage in this form. To the best of our knowledge, no effective and safe fluorine storage materials are known. Both F and Cl have a huge range of industrial applications, which would benefit from such storage materials, especially if they can achieve high storage capacity, stability and reversibility.

In this work, we found that a series of CsF*_n_* (*n* = 1, 2, 3, 5) compounds can be stable at zero temperature and ambient pressure. One mole of CsF_5_ (227.9 g, occupying the volume of 0.07 L) contains 2 moles of F_2_ gas (which in the free state would occupy the volume of 44.8 L - hence, storage in the form of CsF_5_ is three orders of magnitude more efficient, and much safer, than in the form of pure F_2_ gas). The reaction CsF_5_ = CsF + 2F_2_(gas), is thermodynamically unfavourable at zero temperature (the enthalpy of this reaction is 88.41 kJ/mol), but will be favourable on increasing temperatures, due to the higher entropy of the F_2_ gas (202.8 J/(mol·K) at standard conditions)^16^. The calculated thermodynamic properties of these defluorination reactions are given in Table I. It can be seen that such compounds as CsF_3_ can be thermally decomposed, and then again be synthesized at lower temperatures at nearly room temperature window. CsF_5_, having the highest F content, can be used for fluorine storage at low temperature conditions. Thermodynamic stability of the predicted polyfluorides at atmospheric pressure means that there will be ways to “recharge” them with fluorine after defluorination, and such reversibility is a strong advantage of the proposed fluorine storage materials.

**Figure 6:**
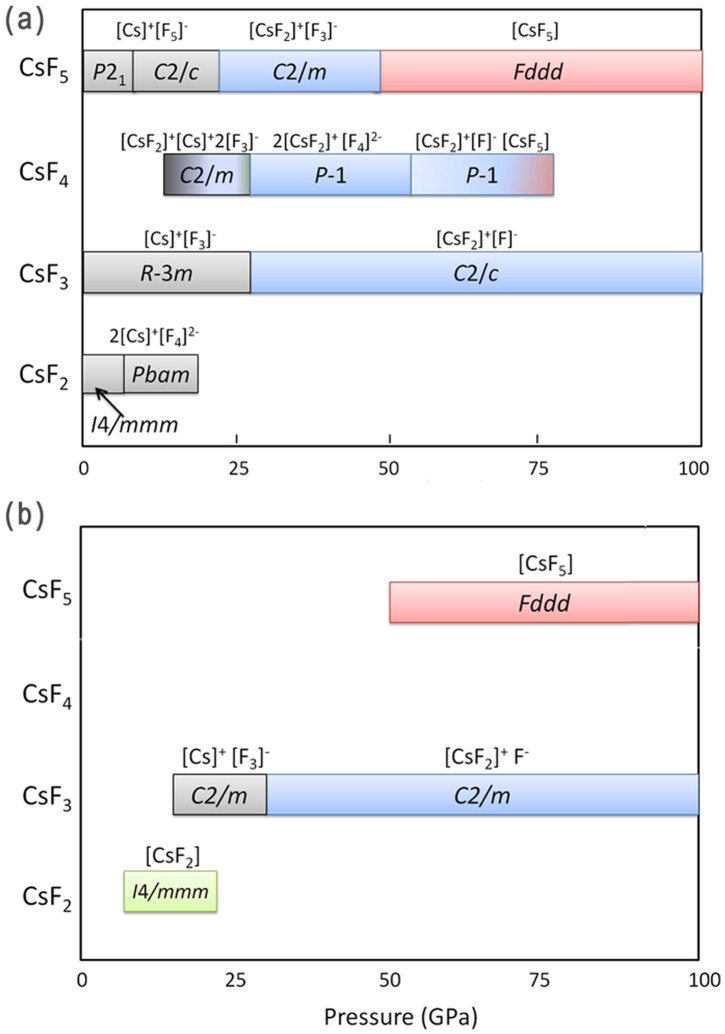
Comparison of CsF*_n_* (*n* = 2,3,4,5) stability phase diagram with respect to pressure. (a) revised results from this study; (b) results from previous study^5^. Note that that each colour represents distinct Cs's valence state in the given compounds (grey: Cs^+^; green: Cs^2+^; blue: Cs^3+^; red: Cs^5+^), while the gradient colour indicate mixed valence states in between. The *I*4/*mmm*-CsF_2_ structure in (a) and (b) are very different. The only common phase between (a) and (b) is *Fddd*-CsF_5_.

We have presented a comprehensive study of possible stable compounds in the CsF-F binary system under pressure. CsF*_n_* phases show extremely rich chemistry. At ambient pressure, novel and unexpected compounds CsF_2_, CsF_3_, CsF_5_ are thermodynamically stable because of the formation of polyfluoride anions of [F_3_]^−^, [F_4_]^2−^, [F_5_]^−^. Our results confirm the previously proposed polyfluoride anions (F_3_^−^, F_5_^−^), and suggest a new ion (F_4_^2−^). Under high pressure, 5*p* electrons of Cs atoms can become chemically active, making Cs^3+^ and Cs^5+^ energetically favourable. Although our prediction found Cs^2+^ and Cs^4+^ states are far from being stable, stochiometric compounds CsF_2_ and CsF_4_ can become stable at 0–20 and 15–80 GPa, respectively, but these contain Cs^+^, Cs^3+^, Cs^5+^, [F_4_]^2−^, [F_3_]^−^. As shown in Fig. 6, crystal structures of caesium polyfluorides can be summarized as packings of Cs-containing cations (Cs^+^, [CsF_2_]^+^), polyfluoride anions ([F_3_]^−^, [F_4_]^2−^, [F_5_]^−^), and neutral molecular species (CsF_5_). Our hope is that this report will stimulate further experimental studies and serve as a guide for the design of fluorine storage materials.

## Methods

Searches for the stable compounds and structures were performed using an evolutionary algorithm, as implemented in the USPEX code^8^,^9^,^10^,^11^. The most significant feature of USPEX we used in this work is the capability of optimizing the composition and crystal structures simultaneously - as opposed to the more usual structure predictions at fixed chemical composition^1^,^14^. The compositional search space is described via building blocks (for example, search for all compositions in a form of [*x*CsF + *y*F]). During the initialization, USPEX samples the whole range of compositions of interest randomly and sparsely. Chemistry-preserving constraints in the variation operators are lifted and replaced by the block correction scheme which ensures that a child structure is within the desired area of compositional space, and a special “chemical transmutation” operator is introduced. Stable compositions are determined using the convex hull construction: a compound is thermodynamically stable if the enthalpy of its decomposition into any other compounds is positive. Structure prediction was done in conjunction with ab initio structure relaxations based on density functional theory (DFT) within the Perdew-Burke-Ernzerhof (PBE) generalized gradient approximation (GGA)^17^ as implemented in the VASP code^18^. For structural relaxation, we used the all-electron projector-augmented wave (PAW) method and the plane wave basis set with the 600 eV kinetic energy cutoff; the Brillouin zone was sampled by uniform Gamma-centered meshes with the resolution 2*π* × 0.06 Å^−1^. For post-processing, the selected low-enthalpy structures were treated by using hard PAW potential of F (F_h), using a energy cut off of 1000 eV. Such calculations provide an excellent description of the known structures (CsF and F_2_) and their energetics. To ensure that the obtained structures are dynamically stable, we calculated phonon frequencies throughout the Brillouin zone using the finite-displacement approach as implemented in the Phonopy code^19^. The vibrational entropies and enthalpies are obtained by directly summing over the calculated phonon frequencies, in order to calculate the free energy (see online supporting information, similar methods have been widely used for simulation of dehydration reactions for hydrogen storage materials^20^,^21^). Charge transfer was investigated on the basis of the electron density using Bader's analysis^22^ as implemented in a grid-based algorithm without lattice bias^23^. Electron localization function (ELF)^24^ is also calculated in order to analyze chemical bonding for the selected compounds.

## Author Contributions

Q.Z. designed the project and carried out structure prediction and electronic structure calculations. Q.-F.Z performed phonon calculations. Q.Z. and A.R.O analysed the data and wrote the paper.

## Supplementary Material

Supplementary Information Supplementary Online Materials: Formation of Stoichiometric CsFn Compounds

## Figures and Tables

**Table 1:** Investigated reactions of the CsF-F system at ambient pressure conditions. wt% gives the weight content of released F_2_ gas. ΔH^0*K*^ and ΔH^300*K*^ are the calculated enthalpies at *T* = 0 K and 300 K, including the vibrational energies in (kJ/mol). ΔS^300*K*^ is the corresponding formation entropy in J/(K·mol). *T_c_* is the predicted decomposition temperature at standard atmosphere (1 bar). Note that F_2_ is treated as crystalline solid at 0 K

Reactions	wt %	ΔH^0*K*^	ΔH^300*K*^	ΔS^300*K*^	*T_c_*(°C)
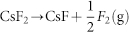	11.1	44.30	37.59	78.25	218
CsF_3_ → CsF + F_2_(g)	20.0	72.24	63.41	152.29	150
CsF_5_ → CsF + 2F_2_(g)	33.3	88.41	76.73	284.96	-15
