# Supplementary material for: Formation of Stoichiometric CsFn Compounds
Source: Sci Rep. 2015 Jan 22;5:7875. doi: 10.1038/srep07875 (PMC4302300; doi:10.1038/srep07875)
Supplement: Supplementary Information — Supplementary Online Materials: Formation of Stoichiometric CsFn Compounds [file srep07875-s1.pdf]

Supplementary Online Materials: Formation of Stoichiometric CsF_n Compounds

Qiang Zhu,[1,a] Artem R. Oganov,[1,2,3] and Qingfeng Zeng[4]

[1] *Department of Geosciences, Stony Brook University, Center for Materials by Design, Institute for Advanced Computational Science, Stony Brook University, NY 11794, USA*

[2] *Department of Problems of Physics and Energetics, Moscow Institute of Physics and Technology, 9 Institutskiy lane, Dolgoprudny city, Moscow Region, 141700, Russia*

[3] *School of Materials Science and Engineering, Northwestern Polytechnical University, Xi'an,710072, China*

[4] *Science and Technology on Thermostructural Composite Materials Laboratory, Northwestern Polytechnical University, Xi'an, 710072, China*

(Dated: 16 June 2014)

I. STABILITY OF CsF UNDER PRESSURE

Our detailed variable-composition structure searches between CsF and F, implicitly assume that CsF should be stable at all pressures. To check this, we also performed structure searches with up to 20 atoms for the entire composition space between Cs and F at 0 and 100 GPa, respectively. As shown in Fig. S1, we found that (1) CsF is stable at both ranges; (2) most of the new stoichiometries are located between CsF and F. This assures that our search strategy is correct.

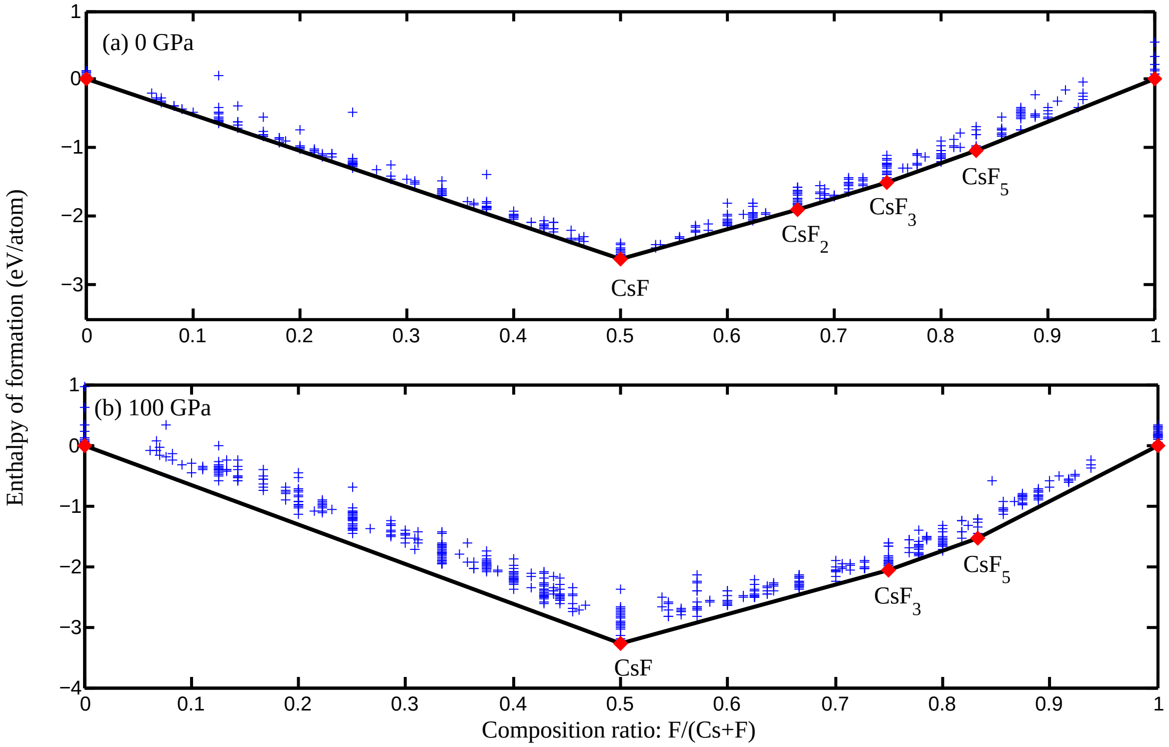

FIG. S1. Relatively stabilities for all compounds in the Cs-F system at (a) 0 GPa; and (b) 100 GPa, respectively.

---

[a] Electronic mail: qiang.zhu@stonybrook.edu

II. VIBRATIONS AND THERMODYNAMICS OF CsF_n AT AMBIENT PRESSURE

A. Free energy of solids

The calculated vibrational behaviors for CsF_n (n=1,2,3,5) compounds under ambient pressures are given in Fig. S2. All of the dispersion curves have no imaginary phonon frequencies, indicating that they are all dynamically stable. The phonon densities of states were also calculated using a 10×10×10 grid of wave vectors.

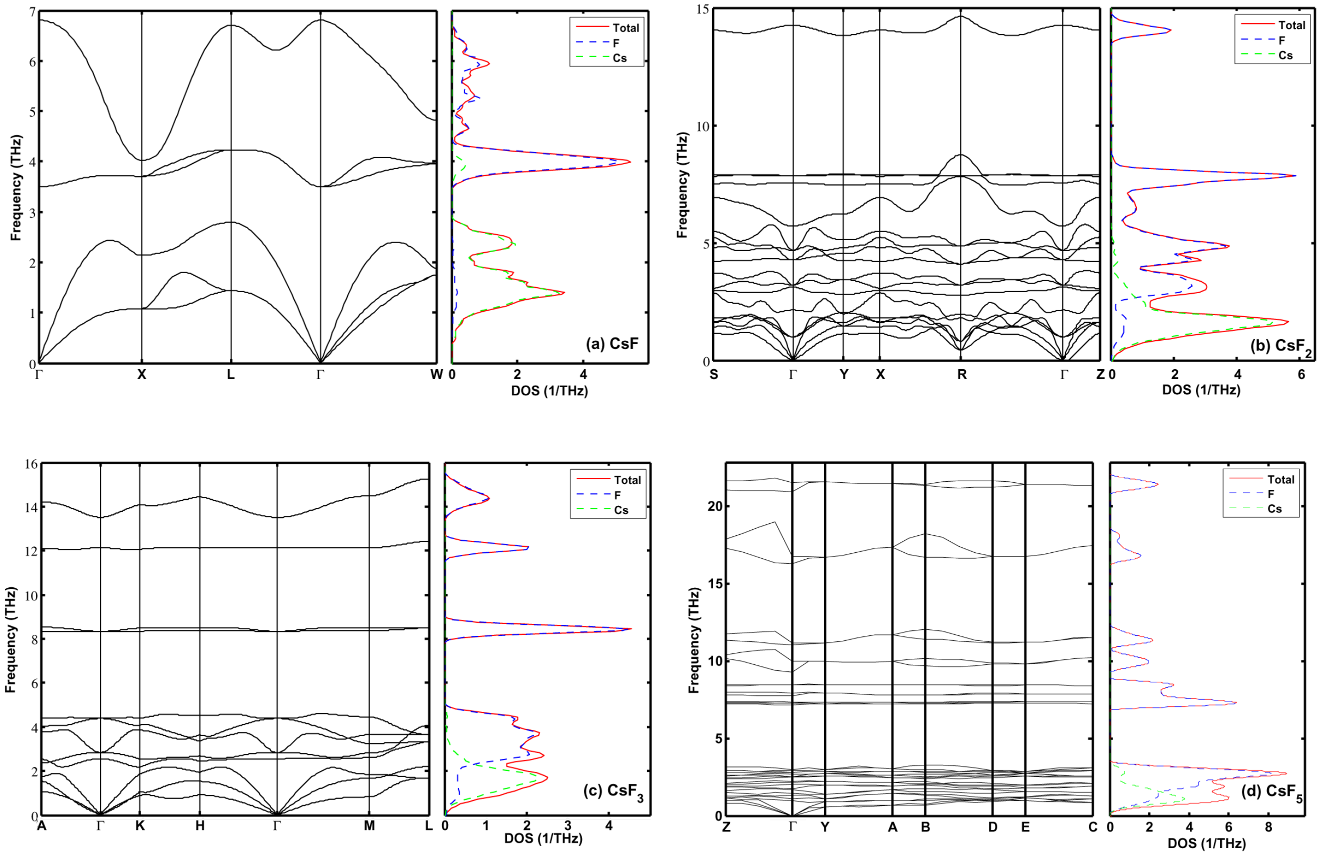

FIG. S2. Calculated phonon dispersion and density of states of (a) $Fm\bar{3}m$-CsF; (b) $I4/mmm$-CsF_2; (c) $R\bar{3}m$-CsF_3; (d) $P2_1$-CsF_5 at ambient pressure.

The free energy of solid (such as CsF_n compounds), can be expressed as,

$$G(T, V) = U(V) + F_{\text{elec}} + F_{\text{vib}}, \tag{1}$$

where U is the internal lattice energy, while F_{elec} and F_{vib} denote the electronic and vibrational contributions. The electronic excitations are usually neglected, while vibrational contributions represents the phonon energy, which can be treated in the harmonic approximation, by summing over the normal modes frequencies,

$$F_{\text{vib}} = \frac{1}{2}\sum_{i=1}^{n} hv_i[\frac{1}{2} + \frac{1}{\exp(-hv_i/kT) - 1}], \tag{2}$$

where k is Boltzmann constant, h is Planck constant.

B. Free energy of F_2 gas

On the other hand, the free energy of diatomic F_2 gas molecule can be expressed as:

$$G(\mathrm{F}_2) = U(\mathrm{F}_2) + \frac{7}{2}k_B T - TS(\mathrm{F}_2), \tag{3}$$

where the entropy S can be divided into there parts, namely, translation entropy S_{trans}, rotational entropy S_{rot}, vibrational entropy S_{vib} and electronic entropy S_{elec}. Again, S_{elec} is neglected, while the other terms can be defined as,

$$\begin{aligned} S_{\mathrm{trans}} &= \frac{5}{2}Nk + Nk\ln[\frac{V}{N}(\frac{2\pi mkT}{h^2})^{3/2}], \\ S_{\mathrm{rot}} &= Nk + Nk\ln\frac{T}{2\Theta_{\mathrm{rot}}}, \\ S_{\mathrm{vib}} &= \frac{Nk\Theta_{\mathrm{vib}}(\frac{1}{2} + \frac{1}{\exp(\Theta_{\mathrm{vib}}/T)-1})}{T} + Nk\ln(\frac{\exp(-\Theta_{\mathrm{vib}}/T)}{1-\exp(-\Theta_{\mathrm{vib}}/T)}). \end{aligned} \tag{4}$$

Here, Θ_{rot} and Θ_{vib} are the characteristic rotational and vibrational temperatures, respectively.

$$\begin{aligned} \Theta_{\mathrm{rot}} &= \frac{(h/2\pi)^2}{2kI}, \\ \Theta_{\mathrm{vib}} &= \frac{hv}{k}, \end{aligned} \tag{5}$$

where I is a molecular moment of inertia, v is the vibration frequency of F_2 molecule. To be consistent with the free energy calculation of solids, here we calculated the entropy of F_2 within the same functional. The relaxed F-F bond length in F_2 molecule is 1.43 Å, in satisfactory agreement with the experimental report (1.42 Å). The theoretical vibrational frequency for F-F stretching modes of F_2 is 30.1 THz, compared with the experimental value 27.489 THz. Note that the vibrational component of the gas entropy here is very small (less than 10% of the total).

The calculated temperature dependent $S(\mathrm{F}_2)$ is shown in Fig. S3. Compared to the experimental value ($S^{\mathrm{expt}}(\mathrm{F}_2)$ = 202.8 J/(mol · K)) at standard conditions, our results are close.

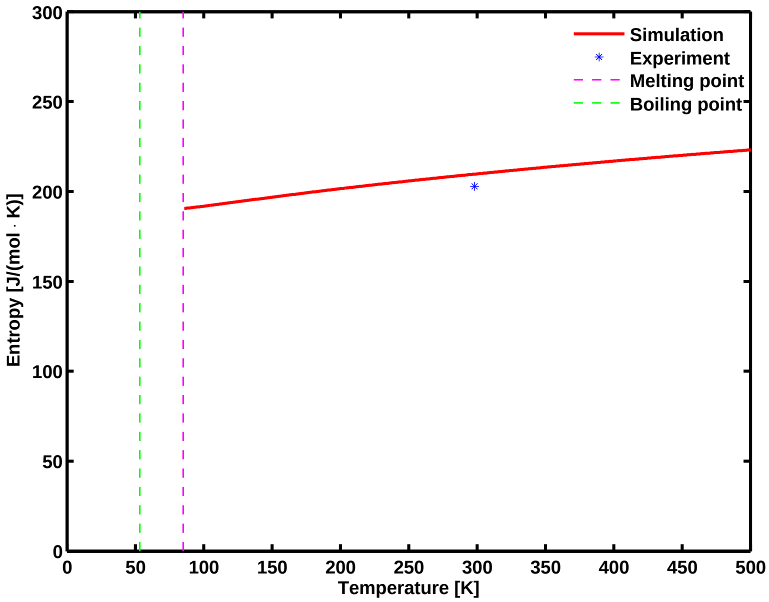

FIG. S3. The calculated entropy of F_2 gas at different temperatures. The melting point (53.48 K) and boiling point (85.03 K) are also shown for being consistent.

C. Thermochemistry of the suggested reactions

By applying the eq. 1 and eq. 3, we obtain the formation energy of the suggested chemical reactions in Table 1. The results are shown in S4. And the predicted decomposition temperatures are 491 K for CsF_2, 423 K for CsF_3, and 258 K for CsF_5, respectively. All of these temperatures are easily accessible, thus these materials are promising for reversible fluorine storage materials.

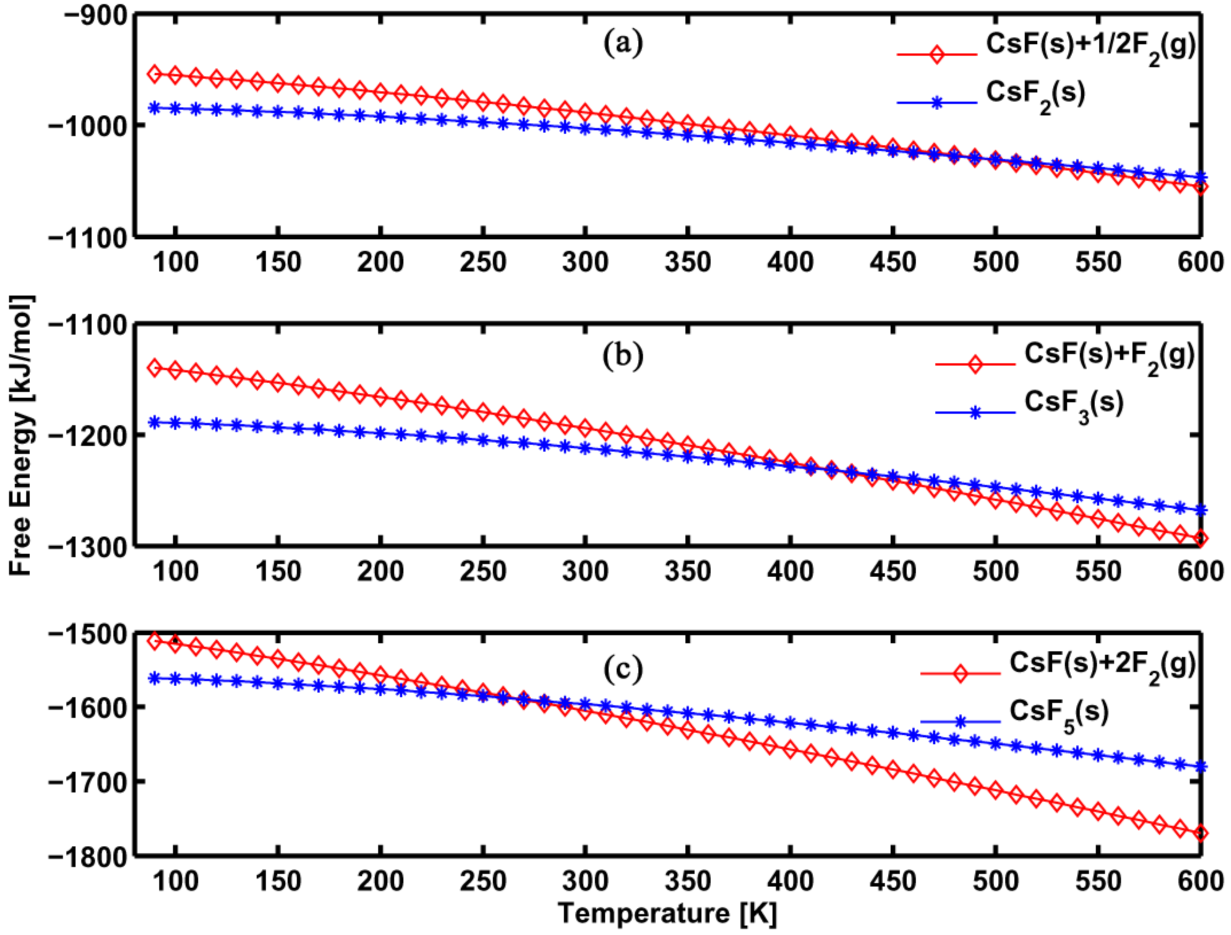

FIG. S4. The temperature dependence of free energy of the suggested chemical reactions (a)$CsF_2 \rightarrow CsF + \frac{1}{2}F_2(g)$; (b) $CsF_3 \rightarrow CsF + F_2(g)$; (c) $CsF_5 \rightarrow CsF + 2F_2(g)$.

III. STABILITY OF CsF_n (n>1) COMPOUNDS UNDER PRESSURE

The detailed results for variable-composition structure searches between CsF and F, are shown in Fig. S5. There is clearly a general trend that the formation enthalpies of CsF_n (n≥1) compounds decrease with pressure, suggesting that the Cs atoms can be oxidized further.

For the predicted stable high pressure phases, we calculated phonon frequencies throughout the Brillouin zone using the finite-displacement approach as implemented in the Phonopy code. As shown in Fig. S6, the absence of imaginary frequencies ensures that the obtained structures are dynamically stable.

IV. CRYSTALLOGRAPHIC DATA

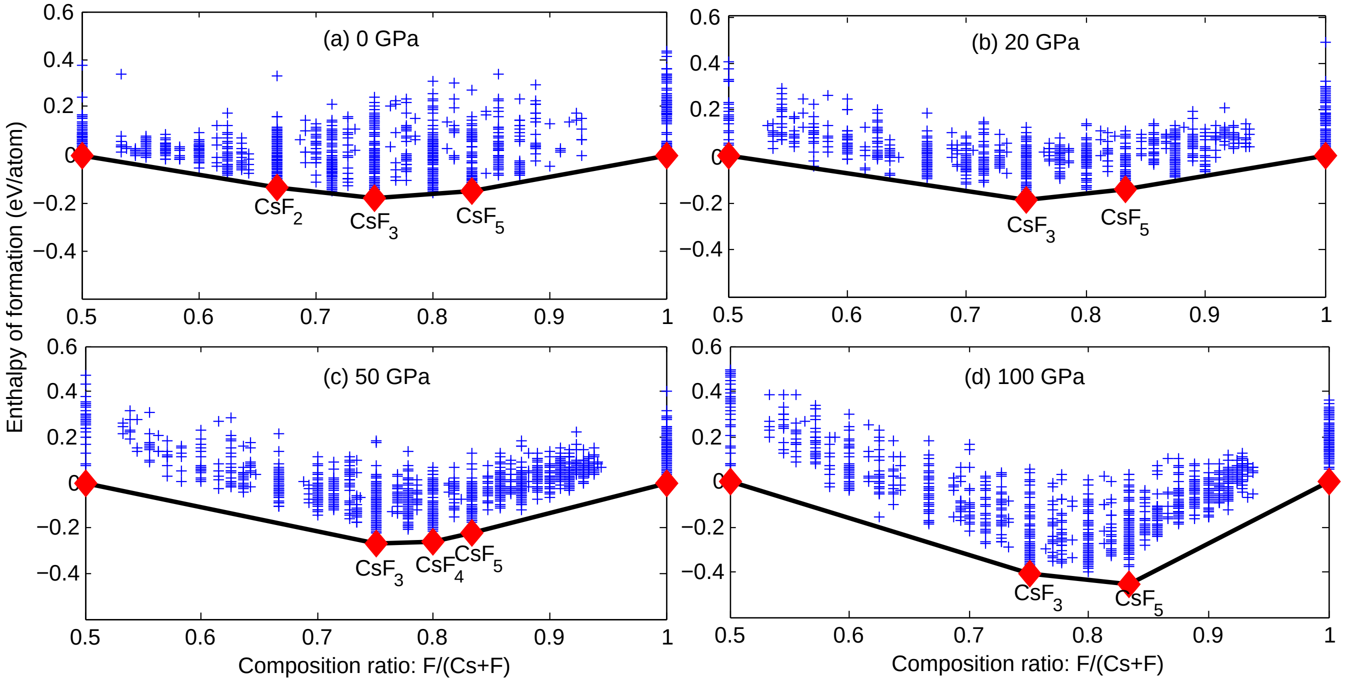

FIG. S5. Relatively stabilities for all compounds in the CsF-F system at (a) 0 GPa; (b) 20 GPa; (c) 50 GPa; and (d) 100 GPa, respectively.

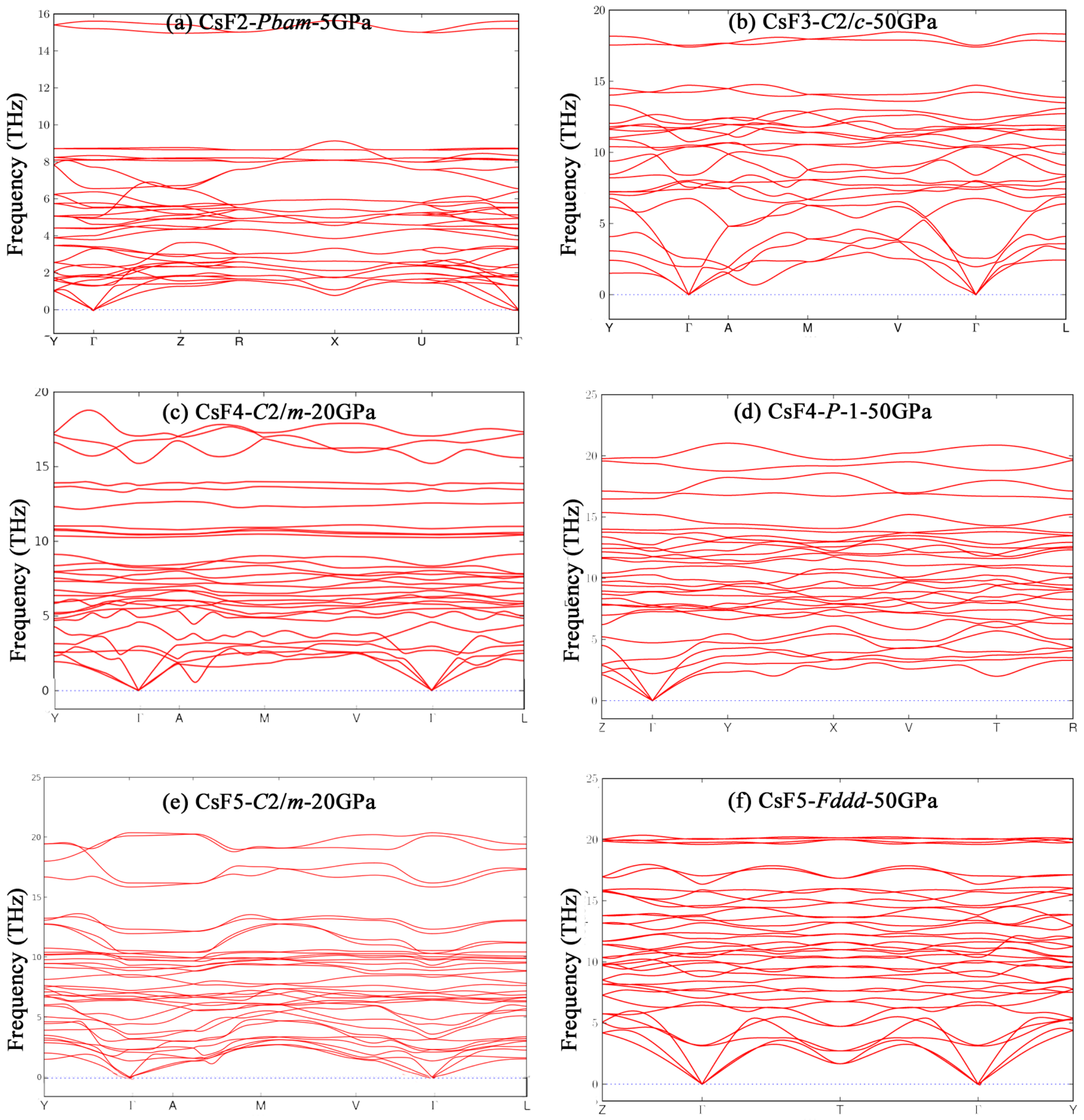

FIG. S6. Phonon dispersion curves of CsF$_n$ compounds (a) $Pbam$-CsF$_2$ at 5 GPa; (b) $C2/c$-CsF$_3$ at 50 GPa; (c) $C2/m$-CsF$_4$ at 20 GPa; (d) $P\bar{1}$-CsF$_4$ at 50 GPa; (e) $C2/m$-CsF$_5$ at 20 GPa; and (f) $Fdddm$-CsF$_4$ at 50 GPa respectively.

TABLE S1. The detailed crystal structures of CsF_n compounds at different pressures.

Phase	Pressure (GPa)	Space group	Lattice parameters	Atomic coordinates
CsF_2	0	$I4/mmm$	a= 4.111 Å	Cs(4e) 0.000 0.000 0.861
			c=16.163 Å	F1(4e) 0.000 0.000 0.682
				F2(4e) 0.000 0.000 0.554
CsF_2	10	$Pbam$	a= 7.862 Å	Cs(4g) 0.167 0.666 0.000
			b= 7.365 Å	F1(4h) 0.039 0.107 0.500
			c= 3.635 Å	F2(4h) 0.372 0.864 0.500
CsF_3	0	$R\bar{3}m$	a= 5.455 Å	Cs(3a) 0.000 0.000 0.000
			c= 9.415 Å	F1(3b) 0.000 0.000 0.500
				F2(6c) 0.000 0.000 0.315
CsF_3	100	$C2/c$	a= 6.322 Å	Cs(4c) 0.250 0.250 0.000
			b= 3.692 Å	F1(4e) 0.000 0.163 0.250
			c= 6.382 Å	F2(8f) 0.612 0.172 0.119
			β=100.65°	
CsF_4	20	$C2/m$	a= 7.207 Å	Cs1(2a) 0.000 0.000 0.000
			b= 4.609 Å	Cs2(2b) 0.000 0.000 0.500
			c= 8.724 Å	F1(4i) 0.189 0.000 0.607
			β=58.43°	F2(4i) 0.417 0.000 0.215
				F3(4i) 0.429 0.000 0.611
				F4(4i) 0.756 0.000 0.866
CsF_4	50	P-1	a= 6.064 Å	Cs1(1a) 0.000 0.000 0.000
			b= 4.101 Å	Cs2(1b) 0.500 0.500 0.500
			c= 4.031 Å	F1(2i) 0.527 0.780 0.913
			α=88.68°	F2(2i) 0.197 0.629 0.979
			β =84.76°	F3(2i) 0.269 0.980 0.448
			γ=92.32°	F4(2i) 0.916 0.645 0.515
CsF_4	70	P-1	a= 6.732 Å	Cs1(2i) 0.858 0.070 0.244
			b= 3.814 Å	Cs2(2i) 0.604 0.356 0.751
			c= 7.128 Å	F1(2i) 0.086 0.738 0.496
			α=97.63°	F2(2i) 0.863 0.153 0.972
			β =88.30°	F3(2i) 0.964 0.530 0.792
			γ=89.31°	F4(2i) 0.444 0.786 0.585
				F5(2i) 0.526 0.215 0.103
				F6(2i) 0.737 0.680 0.073
				F7(2i) 0.253 0.301 0.609
				F8(2i) 0.236 0.081 0.310
CsF_5	0	$P2_1$	a= 5.858 Å	Cs(2a) 0.815 0.000 0.740
			b= 8.235 Å	F1(2a) 0.777 0.234 0.232
			b= 5.184 Å	F2(2a) 0.744 0.603 0.505
			β= 106.67°	F3(2a) 0.220 0.267 0.764
				F4(2a) 0.286 0.972 0.283
				F5(2a) 0.517 0.250 0.014
CsF_5	10	$C2/c$	a= 7.794 Å	Cs(4e) 0.000 0.801 0.250
			b= 4.440 Å	F1(4e) 0.000 0.342 0.250
			c= 3.613 Å	F2(8f) 0.327 0.013 0.719
			β= 79.81°	F3(8f) 0.679 0.382 0.708
CsF_5	30	$C2/m$	a= 8.338 Å	Cs(2a) 0.000 0.000 0.000
			b= 4.314 Å	F1(2c) 0.000 0.000 0.500
			b= 5.292 Å	F2(4i) 0.345 0.000 0.497
			β= 42.02°	F3(4i) 0.714 0.000 0.926
CsF_5	100	$Fdd2$	a=13.726 Å	Cs(8a) 0.000 0.000 0.228
			b= 5.357 Å	F1(8a) 0.000 0.000 0.853
			c= 5.146 Å	F2(16b) 0.576 0.146 0.022
				F3(16b) 0.162 0.499 0.346
